# Extracellular Alpha-Synuclein Promotes a Neuroinhibitory Secretory Phenotype in Astrocytes

**DOI:** 10.3390/life10090183

**Published:** 2020-09-08

**Authors:** Bruno Di Marco Vieira, Rowan A. W. Radford, Junna Hayashi, Emma D. Eaton, Ben Greenaway, Mark Jambas, Eugen B. Petcu, Roger S. Chung, Dean L. Pountney

**Affiliations:** 1 School of Medical Science, Griffith University, Gold Coast 4222, Australia; bruno.vieira@mail.mcgill.ca (B.D.M.V.); junna.hayashi@anu.edu.au (J.H.); ben.greenaway@griffithuni.edu.au (B.G.); mark.jambas@gmail.com (M.J.); 2 Department of Biomedical Sciences, Faculty of Medicine & Health Sciences, Macquarie University, Sydney 2109, Australia; rowan.radford@mq.edu.au (R.A.W.R.); roger.chung@mq.edu.au (R.S.C.); 3 Menzies Research Institute Tasmania, University of Tasmania, Hobart 7000, Australia; emma.eaton@utas.edu.au; 4 School of Medicine, Griffith University, Gold Coast 4222, Australia; e.petcu@griffith.edu.au

**Keywords:** astrocytes, astrogliosis, MSA, DLB, Munc18

## Abstract

Multiple system atrophy (MSA) and dementia with Lewy bodies (DLB) are α-synucleinopathies that exhibit widespread astrogliosis as a component of the neuroinflammatory response. Munc18, a protein critical to vesicle exocytosis, was previously found to strongly mark morphologically activated astrocytes in brain tissue of MSA patients. Immunofluorescence of MSA, DLB and normal brain tissue sections was combined with cell culture and co-culture experiments to investigate the relationship between extracellular α-synuclein and the transition to a secretory astrocyte phenotype. Increased Munc18-positive vesicles were resolved in activated astrocytes in MSA and DLB tissue compared to controls, and they were also significantly upregulated in the human 1321N1 astrocytoma cell line upon treatment with α-synuclein, with parallel increases in GFAP expression and IL-6 secretion. In co-culture experiments, rat primary astrocytes pretreated with α-synuclein inhibited the growth of neurites of co-cultured primary rat neurons and upregulated chondroitin sulphate proteoglycan. Taken together, these results indicate that the secretory machinery is significantly upregulated in the astrocyte response to extracellular α-synuclein and may participate in the release of neuroinhibitory and proinflammatory factors in α-synucleinopathies.

## 1. Background

Abnormal aggregates of alpha-synuclein (α-syn) constitute the common histopathological hallmark of neurodegenerative disorders such as Parkinson’s disease (PD), multiple systems atrophy (MSA) and dementia with Lewy bodies (DLB); all grouped as α-synucleinopathies [1,2,3,4]. Abundant as disordered monomer or alpha-helical tetramer in the presynaptic region of neurons [5], α-syn is a protein that, for reasons yet unknown, misfolds as oligomers, fibrils and protofibrils; which are major components of intracellular inclusion bodies known as Lewy bodies (LBs), Lewy neurites (LNs) or glial cytoplasmic inclusions (GCIs) [6,7]. In addition to misfolding, α-syn may be released and taken up by surrounding cells, as evidenced in experiments involving cell lines, rodent models and human tissue grafts [8,9,10]. Importantly, the direct action of α-syn on astrocytes is known to induce changes of morphology, transcription of target genes and secretion of proinflammatory cytokines that characterize neuroinflammation [11,12,13,14]. This process involves, but is not restricted to, stimulation (in microglia) or induction (in astrocytes) of toll-like receptors (TLR 4 and 2), activation of effectors that translocate to the nucleus and modulation of the glial transcriptome [15,16]. Phosphorylation of p38 MAPK and JNK [17], and nuclear translocation of NF-κB in B-cells are some of the signaling pathways affected; with the induction of proinflammatory factors, such as Cox-2, iNOS, TNF-α, Il-1ß and IL-6 [18,19]. At the microscopic level, increased transcription of the intermediate filaments vimentin and glial fibrillary acidic protein (GFAP) leads to the typical hypertrophy and arborization that characterize reactive astrocytes [20].

The most numerous glial cells, astrocytes, are known to actively participate in functions that include signal integration and secretion of both regenerative and damaging factors, as evidenced by studies in neurovascular coupling, synaptic pruning, neurotransmitter recycling and calcium-dependent exocytosis [21,22,23,24,25,26,27]. Importantly, to allow vesicle trafficking, all eukaryotic cells utilize molecular machinery that catalyzes membrane fusion that includes the SNAREs (soluble NSF-attachment protein receptors) and SM (Sec1/Munc18-like) proteins [28,29,30]. One of the SM proteins, Munc18, also known as syntaxin-binding protein 1 (STXBP1), which is expressed abundantly in neurons, glia and neuroendocrine cells, plays a central role in docking, priming and decreasing the rate of ectopic interactions of its main SNARE partner syntaxin [31,32,33,34]. 

Previously, we have shown that immunostaining of Munc18 was higher in astrocytes in affected brains of patients with MSA [35]. Here, we further characterized brain tissue and cellular phenomena related to astrocyte activation in α-synucleinopathies. We have conducted in vitro studies showing that exogenous α-syn treatment increased the number of Munc18-positive vesicles in human 1321N1 astrocytes and we resolved punctate immunostaining attributed to Munc18-positive vesicles that are increased in brains of MSA and DLB cases, when compared to age-matched controls. Thus, both in vitro and post-mortem studies implicate Munc18 in astrocyte reactivity that is α-syn dependent. Furthermore, in cell culture experiments, human 1321N1 astrocytes treated with exogenous α-syn showed increased GFAP expression, activated morphology and increased IL-6 secretion. Moreover, rat primary astrocytes pretreated with α-synuclein inhibited the growth of neurites of co-cultured primary rat neurons and secreted chondroitin sulphate proteoglycan (CSPG). Overall, the results indicate that extracellular α-syn acts on astrocytes to promote a secretory, neuroinhibitory phenotype.

## 2. Methods

### 2.1. Immunohistochemistry

Human brain tissue of 4 DLB (80 ± 7 years, post-mortem interval (PMI) 8 ± 3), 4 MSA (70 ± 7 years, PMI 8 ± 3 h), and 4 normal cases (74 ± 9 years; PMI, 14 ± 8 h) was obtained from the South Australian Brain Bank including five different anatomical regions: frontal cortex, temporal cortex, motor cortex, visual cortex and hippocampus. Formalin-fixed, paraffin-embedded sections (5 μm) underwent heat-induced antigen retrieval in 1 mM EDTA (pH 8, at 100 °C), followed by blocking with 20% normal horse serum (NHS). Primary rabbit anti-Munc18-1 (Sapphire Biosciences, Ab3451, 1:100) and mouse anti-α-syn (Abcam, LB509, 1:500) antibodies diluted in 1% NHS were incubated overnight, washed, then incubated with Alexa Fluor secondary antibodies (Invitrogen, A21202, A27034, A10042, all 1:200 in 1% NHS in TBS) in the dark for 90 min. Finally, slides were mounted in DAPI Pro-long gold (Life Technologies, Melbourne, Australia), and equivalent anatomical regions were imaged in diseased and control cases. Munc18 vesicles were counted in de-identified images as discrete puncta with Munc18 staining at least five-fold over background.

### 2.2. Cell Culture and Immunocytochemistry

1321N1 cells were cultured in DMEM (Life Technologies, Melbourne, Australia) supplemented with 10% fetal bovine serum (Sigma, Sydney, Australia) and 1% penicillin/streptomycin (Life Technologies, Melbourne, Australia), and incubated at 37 °C, 5% CO_2_. Cells pretreated with 1 mM dbcAMP [36] for 24 h were seeded into 24 well plates (20,000 cells/well) on 12 mm glass coverslips and allowed to attach overnight. Recombinant α-syn was prepared as described previously [37]. Briefly, human α-syn was expressed in *Escherichia coli* BL21 DE3 transformed with the human α-syn cDNA sequences. α-Syn was purified from cell lysate using a HiPrepTM 16/10 Q FF (GE Healthcare) anion exchange, and monomer was obtained from the peak fraction by gel permeation chromatography (SuperdexTM 75 10/300 GL; GE Healthcare) in 20 mM Tris-HCl, 0.15 M NaCl (pH 7.4), then snap frozen in liquid nitrogen and stored at −20 °C prior to use. Purity was > 95% by Coomassie-stained SDS-PAGE. Protein concentration was determined by Pierce bicinchoninic acid (BCA) kit. α-Syn monomer diluted 1:100 in culture medium was applied to cells at a final concentration of 0.5 µM for 3–24 h. Control treatments contained buffer vehicle only. Treatments with exogenous α-syn were stopped by washing with PBS for 2 min and fixation with 3:1 methanol–acetone. After fixation, cells were blocked with 20% NHS, then washed prior to incubation with primary rabbit antibodies to Munc18 (Sapphire Biosciences, Ab3451, 1:200), GFAP (Invitrogen) and/or α-syn (Abcam; LB509, 1:200) in 1% NHS overnight. After washing, cells were incubated with secondary antibodies (Alexa Fluor; 1:200 in 1% NHS) for 1 h, washed and mounted with DAPI Pro-long gold (Life Technologies, Melbourne, Australia). Negative controls contained only secondary antibodies.

For primary astrocyte cultures, cerebral cortices dissected from postnatal days 1–3 hooded Wistar rats were placed in HBSS medium (Sigma, St. Louis, MO, USA), trypsinized and incubated at 37 °C for 25 min. The medium was replaced with DMEM + 10% fetal calf serum (Invitrogen, Boston, MA, USA) and tissue triturated, cell suspension filtered (0.6 mm gauze), diluted with DMEM-10S, centrifuged (10 min; 500× *g*; 4 °C), the supernatant removed and DMEM-10S added. The triturated cell suspension was cultivated in poly-L-lysine-coated flasks containing DMEM-10S and incubated for 24 h before the media was replaced. The media was then replaced every 48 h until the cells were confluent (8–10 days), then the flask was shaken (250 rpm; 37 °C; 24 h), medium replaced with fresh DMEM-10S supplemented with Ara-C (Sigma, Sydney, Australia) and repeated (48 h) before cells were split. Before experimentation, confluent cultures were maintained in serum-free medium for at least 3 days. Embryonic cortical neurons were dissected from embryonic day 17 hooded Wistar rat embryos, trypsinized and, following three washes with 10 mM HEPES to remove the trypsin, cells were plated directly onto confluent astrocyte cultures pretreated for 24 h with either 0.07 µM A53T α-syn, α-syn wild-type (WT), interferon-γ (IFN-γ) positive control or vehicle at a density of 10,000 cells/well on poly-L-lysine-coated 25 mm glass coverslips [38]. Cells were cultured in neurobasal media, B27 supplement, 0.1 mM glutamine, 200 U/mL gentamycin (Invitrogen). Cells were fixed with 4% paraformaldehyde and immunolabeled for the neuron-specific cytoskeletal protein βIII-tubulin and detected using an AlexaFluor-488 secondary antibody. Images were acquired on an Olympus BL-50 upright microscope using a Magnafire CCD camera. The images were imported into HCA-Vision (CSIRO, Sydney, Australia), and the following parameters were used to identify and measure neurites: neuron body channel = 1, size of prefilter = 6, top hat width = 99, minimum area of neuron body = 225 and minimum radius of neuron body = 7.

### 2.3. Confocal Microscopy

Image capture was by Nikon A1R+ confocal microscope or Zeiss LSM 880 Airyscan. Cells were imaged in z-stacks of 5–6 μm total thickness, each containing an average of 30–40 steps (0.15–0.20 nm each), then compiled into one 2D image for analysis. From each slide, images were obtained from four quadrants, following a clockwise direction. Confocal z-series were performed and analyzed via Velocity Software (Perkin Elmer, Boston, MA, USA). For the MSA tissue, image capture was restricted to white matter regions of visual and frontal cortex. For DLB, images were captured from both grey and white matter. For control brain tissue, images were captured from its equivalent MSA or DLB case region. Secondary antibody only slides were imaged and microscope settings for laser voltage, electronic gain and offset set for background levels.

For the analysis of Munc18 cellular images, a stepwise protocol was set up using Columbus 2.3 software (Perkin Elmer, Boston, MA, USA). Briefly, nuclei were identified based on DAPI, and cell number was determined based on the number of nuclei. Next, cytoplasm was identified based on diffuse cytoplasmic Munc18 immunofluorescence with exclusion of nuclei, with Munc18 vesicles being defined in the cytoplasm only, and carrying an individual accumulated relative signal intensity higher than 20% over the background. Finally, large vesicular bodies (such as components of the ER-Golgi or Trans-Golgi networks not representing vesicles in the exocytosis pathway) were excluded based on cytoplasmic location, large diameter (>2.5 um), and high contrast relative to background signal (>50%). GFAP immunofluorescence cytoplasmic intensity was measured using Columbus 2.3 software (Perkin Elmer, Boston, MA, USA) selecting the cytoplasmic region (outer border: −10%, inner border: 45%).

### 2.4. Western Blotting

1321N1 cultures were washed with ice-cold PBS and harvested in ice-cold lysis buffer (20 mM Tris-HCl pH 7.2, 150 mM NaCl, 1% Triton X-100, protease inhibitor cocktail). After 1 h incubation on ice, the cell suspension was centrifuged (20,000× *g*, 5 min), and the supernatant was used for colorimetric protein quantification (Pierce BCA protein assay kit), and 20 μg from each sample was resolved by 10% SDS-PAGE for immunoblotting (Bio-Rad, California). Proteins were then transferred to nitrocellulose membranes, blocked for 1 h with 5% skim milk in TBS with 0.05% Tween-20, and incubated with Munc18 primary antibody (Sapphire Biosciences, Ab3451, 1:1000) overnight at 4 °C. Next, the membranes were washed and incubated for 1 h with antirabbit IgG-HRP secondary antibody (Sigma, 1:10,000 in blocking buffer). Next, developing imaging solutions were prepared (dH_2_O, Luminol, Coumaric, 1 M Tris pH 8.5; and dH_2_O, 30% H_2_O_2_, 1 M Tris pH 8.5) and added to the membranes. Finally, images were captured using a chemiluminescence detection system (ChemiDoc MP, Bio-Rad, Hercules, CA, USA).

### 2.5. ELISA Assays

GFAP and CSPG ELISA assays were conducted as described [36]. Briefly, primary astrocytes were plated into 24 well plates at a density of 10,000 cells/well in DMEM-10S. After 3–4 days, serum-free medium was applied to the cultures for 3 days, after which cells were treated with α-syn or vehicle for 24 h. Cells were then fixed with 4% paraformaldehyde, washed with PBS-0.1% triton-X, quenched with 0.3% H_2_O_2_. They were then blocked with 1% BSA in PBS for 30 min, followed by incubation with polyclonal rabbit anti-GFAP (1:1000; DAKO) or polyclonal rabbit anti-CSPG (1:500; Santa Cruz) and secondary antirabbit HRP-conjugated (1:2000) antibodies for 1 h each. HRP was detected using the TMB-detection system (KPL). For IL-6 ELISA assays, 1321N1 cells were seeded into a 96-well plate and allowed to attach overnight. Cells were then treated with recombinant monomeric α-syn at 0.25 µM for 3 h. An IL-6 human ELISA detection kit was used (Abcam, ab46027) and the assay protocol was followed. Briefly, 100 µL of each IL-6 standard (0 pg/mL, 12.5 pg/mL, 25 pg/mL, 50 pg/mL, 100 pg/mL and 200 pg/mL) was added to the ELISA kit microplate wells. 100 µL of medium from the α-syn treated cells was added to the microplate wells, then biotinylated anti-IL-6 was added, incubated for 1 h at room temperature, washed three times and streptavidin-horseradish peroxidase was added and incubated at room temperature for 30 min, with detection by TMB. Absorbance of the wells was read at 450 nm (BMG FLUOstar OPTIMA Microplate Reader).

### 2.6. Statistical Analysis

For statistical analysis, Student’s t-test was used to compare the means between treated and control groups using GraphPad Prism 6 (by GraphPad Software Inc., San Diego, CA, USA). For neurite outgrowth analysis, the average total neurite outgrowth of each cell was calculated from more than 200 neurons per experiment. Statistical analysis was by multivariate ANOVA (SPSS 16.0, IBM, New York, NY, USA).

## 3. Results

### 3.1. α-Syn Treatment Promotes Increased Astrocyte Expression of Glial Fibrillary Acidic Protein and Chondroitin-Sulphate Proteoglycans and IL-6 Secretion

**Figure 1:**
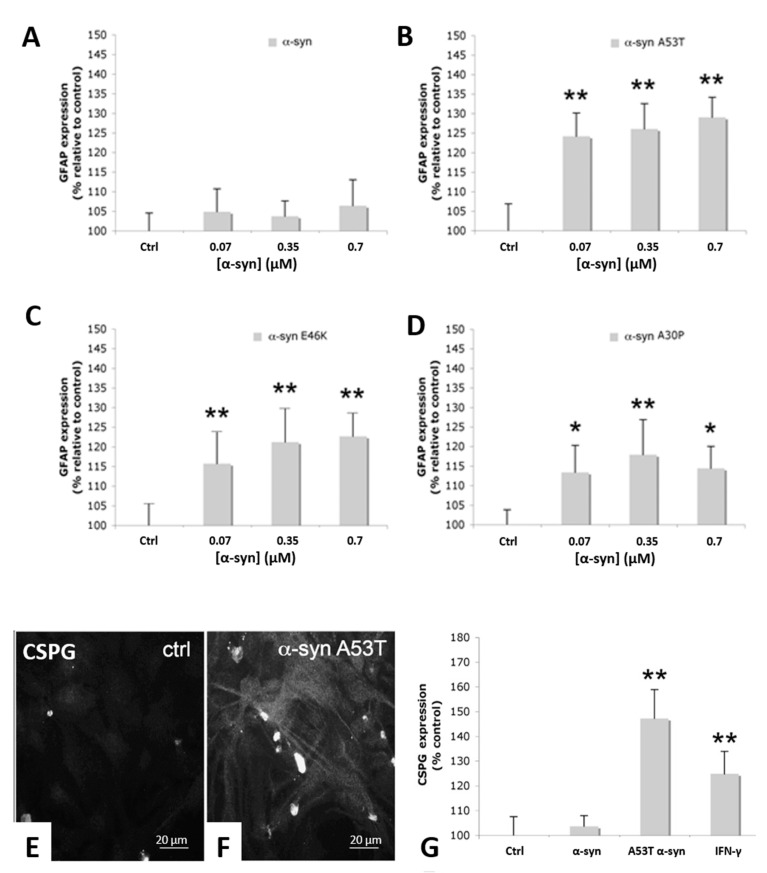
Primary rat astrocyte increased expression of GFAP and CSPG upon α-syn treatments. (**A**–**D**) WT and mutant α-syn treatment and GFAP expression in primary rat astrocytes. GFAP expression in primary rat astrocytes was measured in controls and compared to different α-syn forms tested at three concentrations. (**A**) WT α-syn compared to vehicle-only control. (**B**) Mutant A53T α-syn compared to vehicle-only control. (**C**) Mutant A46K α-syn compared to vehicle-only control. (**D**) Mutant A30P α-syn compared to vehicle-only control. (**E**,**F**) CSPG immunofluorescence of rat primary astrocytes treated with exogenous mutant A53T α-syn and (**F**) vehicle-only control. (**G**) CSPG ELISA assays of primary rat astrocytes treated with exogenous WT α-syn, mutant A53T α-syn, vehicle-only control and IFN-γ positive control. **, *p*, 0.01; *, *p*, 0.05.

Previously, we have reported that treatment of primary rat astrocytes with exogenous recombinant human α-syn monomer can induce an activated phenotype [35]. While both WT and mutant α-syn resulted in morphological changes and increased ERK1/2 phosphoryation in our previous studies, a significant increase in GFAP immunofluorescence indicative of activation was only observed in cells treated with the PD-linked A53T α-syn mutant, whereas MSA and DLB are rarely associated with α-syn gene mutations. Here, we furthered our investigation using A53T, E46K and A30P α-syn mutant proteins in comparison with WT α-syn using an ELISA specific to GFAP. Figure 1A–D shows that, in each case, the mutant α-syn proteins caused significant GFAP upregulation in primary rat astrocytes compared to vehicle-only control. Although there was an increase in GFAP expression observed also with WT α-syn, this did not reach statistical significance. An apparent dose dependence was observed with WT α-syn, A53T and E46K mutants, but this was not statistically significant. The potency of the mutant α-syn forms to induce GFAP expression in astrocytes was in the order of A53T > E46K > A30P > WT.

Morphological changes typical of reactive astrocytes within the injured or diseased brain are generally associated with the expression of growth inhibitory molecules, of which chondroitin-sulphate proteoglycans (CSPGs) are the major family expressed by reactive astrocytes [39]. We, therefore, investigated whether mutant α-syn alters expression of CSPGs by astrocytes. Exogenous A53T α-syn treatment induced significant upregulation in CSPG expression in rat astrocytes after 24 h (Figure 1E–G) compared to both vehicle-only control and exogenous WT α-syn treated astrocytes as determined both by immunofluorescence microscopy (Figure 1E,F) and ELISA assay (Figure 1G).

**Figure 2:**
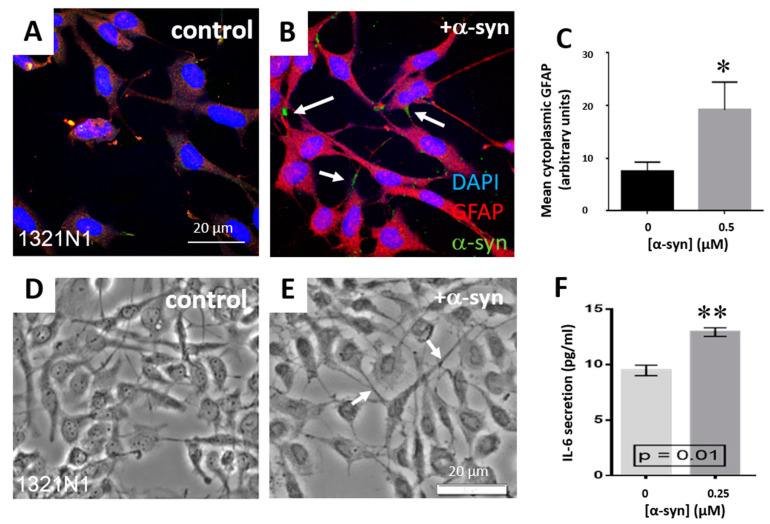
Human 1321N1 astrocytes treated with WT α-syn show increased GFAP expression, activated morphology and IL-6 secretion. (**A**,**B**) Vehicle-treated and α-syn-treated 1321N1 cells. (**C**) GFAP immunofluorescence mean cytoplasmic intensity. (**D**,**E**) DIC images of control and α-syn-treated 1321N1 cells. (**F**) IL-6 concentration in 1321N1 conditioned medium with and without α-syn treatment. Error bars represent ± SEM. **, *p*, 0.01; *, *p*, 0.05.

We hypothesized that α-syn treatments using the human protein may have reduced potency towards non-human cell types. Therefore, we conducted treatments of the human 1321N1 astrocytoma cell line as a model for human astrocytes. Treatment of 1321N1 astrocytoma cells with exogenous WT α-syn led to a statistically significant (*p*, 0.05) increase in GFAP immunofluorescence (Figure 2A–C) and α-syn aggregates were observed at the cell periphery (Figure 2B, arrows). α-Syn treated cells displayed extended and thicker processes by differential interference contrast microscopy (Figure 2D,E arrows), which was similar to the morphology of activated astrocytes observed in MSA tissue by GFAP immunofluorescence [35].

1321N1 astrocytoma cells were incubated with α-syn to determine the effect on IL-6 secretion (Figure 2F). 1321N1 cells treated with α-syn for 3 h had a mean IL-6 secretion of 12.93 pg/mL (SEM = 0.39, *n* = 3), while the control untreated cells had a mean secretion level of 9.47 pg/mL (SEM = 0.47, *n* = 3). This was a statistically significant difference in IL-6 secretion level (*p* = 0.01).

### 3.2. Munc18-Positive Vesicles Are Increased in MSA and DLB Astrocytes

**Figure 3:**
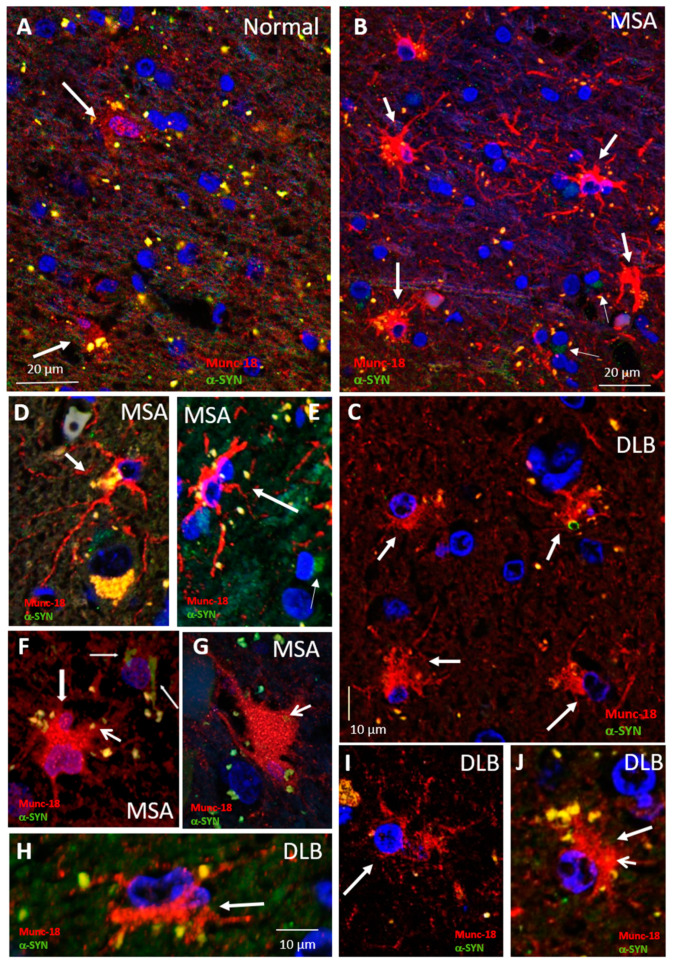
Munc18-positive vesicles in astrocytes in dementia with Lewy bodies (DLB), multiple system atrophy (MSA) and normal tissue. (**A**–**C**) Representative images of Munc18 (**red**) and α-syn (green) immunofluorescence in normal tissue (**A**), MSA (**B**) and DLB (**C**), showing high expression in MSA and DLB tissue compared to normal tissue (frontal cortex) and intense perinuclear punctate expression (normal arrow) in the vicinity of glial cytoplasmic inclusions (GCIs) (small arrows). (**D**,**E**) Enlarged views of MSA tissue. DAPI (**blue**) nuclear stain. (**F**,**G**) Example Munc18 (red) expression in MSA visual cortex, illustrating the intense punctate expression (thick arrow) in activated astrocytes observed in areas of high GCI (normal arrows) pathology. (**H**–**J**) Enlarged views of DLB tissue.

Previously, we reported increased expression of the endomembrane vesicle-associated protein, Munc18, in activated astrocytes in MSA tissue [35]. We further investigated Munc18 in brain tissue of DLB, MSA and controls to determine if increased Munc18 expression was associated with an increase in cytoplasmic vesicles. Munc18-specific immunofluorescence and confocal imaging were used along with α-syn antibodies to allow correlation of Munc18-positive vesicles with α-syn inclusions. The analysis criteria included identification of cells by cell morphology and correlation with immunostaining of α-syn aggregates in the vicinity of the region of interest. Tissue sections from four normal control cases were analyzed. The overall staining from each of the four anatomical regions (motor cortex, frontal cortex, temporal cortex and hippocampus) did not differ significantly between cases with Munc18 staining of individual cells not prominently visible for most cells, but with occasional cells with glial morphology being weakly immunopositive with granular immunofluorescence (Figure 3A). Brain tissue of four cases of MSA were immunostained for Munc18 and α-syn, and two different anatomical regions were examined (frontal and visual cortex) and compared to equivalent regions from the control group. As shown in Figure 3B, α-syn aggregates (GCIs) were localized primarily in the perinuclear region of glial cells (Figure 1B, small arrows). Frequent glial cells displaying the morphology of activated astrocytes were observed close to GCIs that were filled with Munc18-positive vesicles (Figure 3B–E). Munc18-positive vesicles were resolved throughout the cell body and extended processes (Figure 3D–G), but they were insufficiently resolved from each other to count individual vesicles. Brain tissue sections from four cases of DLB were immunostained for Munc18 and α-syn. Figure 3C illustrates the typical images from sections of DLB cases. There is a rich presence of Munc18-positive vesicles, again associated with activated astrocytes, which was clearly increased compared to normal tissue sections, with frequent well-resolved cytoplasmic puncta attributed to secretory vesicles. Occasional colocalization was observed between Munc18 puncta and α-syn. Activated astrocytes with Munc18 staining were less prominent than in the MSA cases. Astrocytic Munc18-positive vesicles were more clearly resolved in the DLB cases, and manual counting of clearly resolved cytoplasmic puncta in perspective views of 3D reconstructions (Figure 3H–J) revealed 50 ± 10 vesicles per cell (*n* = 10), compared to a maximum of 11 resolved Munc18 puncta observed in occasional cells with astrocyte morphology that were weakly stained for Munc18 in the normal cases. As Munc18 is a generalized marker for a wide array of cytoplasmic vesicles, the increased number of Munc18-positive vesicles in astrocytes close to α-syn deposits may indicate upregulation of the secretory pathway.

### 3.3. α-Syn Treatment Promotes Astroglial Accumulation of Munc18-Positive Vesicles

**Figure 4:**
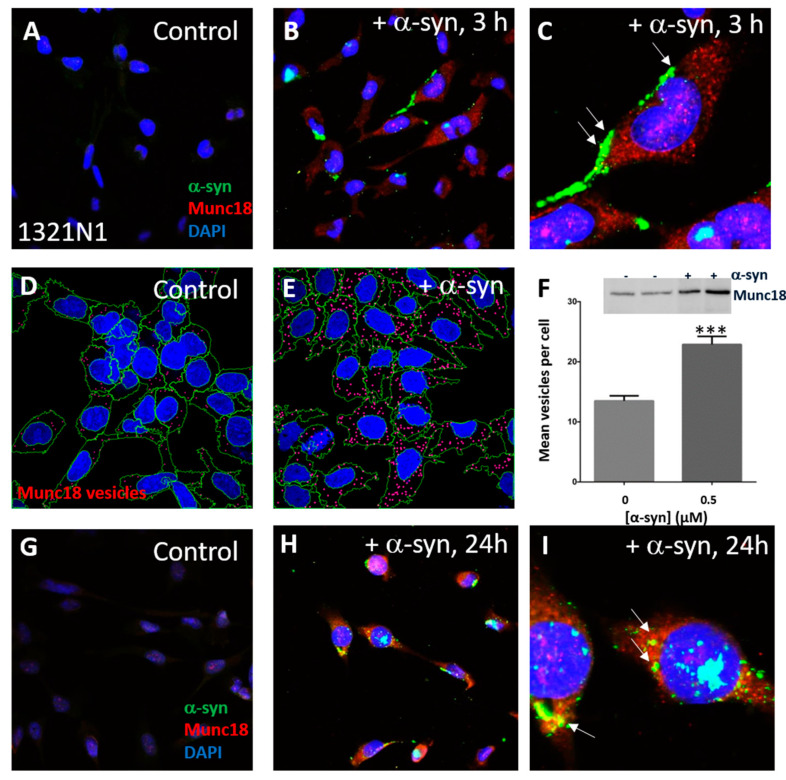
Munc18-positive vesicles increased in α-syn-treated 1321N1 cells. (**A**,**B**) Representative immunofluorescence of control vehicle-only and α-syn-treated 1321N1 cells at 3 h. (**C**) Enlarged area. (**D**,**E**) Representative image outputs from Columbus software showing an increase of Munc18-positive vesicles (in red) in the α-syn-treated group of cells at 3 h. Vesicle positivity is established based on inclusion criteria (cytoplasmic location and signal intensity). The nuclear region is not considered for analysis of Munc18. Red dots are Munc18 vesicles. The nuclear region is blue and cytoplasm is outlined in green. (**F**) Graph of means ± SEM of Munc18-positive vesicles between controls and α-syn treated groups at 3 h. A significant increase (1.7-fold) in the number of positively marked vesicles is observed in the treated group when compared to controls. (**G**,**H**) Representative immunofluorescence of control vehicle-only and α-syn-treated 1321N1 cells at 24 h. (**I**) Enlarged area. ***, *p*, 0.001.

Munc18 is present in virtually all eukaryotic cells, including brain cells and the 1321N1 cell line, and is a generalized marker for endomembrane vesicles. In order to determine the effect of α-syn on Munc18-positive vesicles, 1321N1 cells were treated with exogenous human recombinant α-syn protein (0.5 µM) with granular α-syn accumulations at the cell periphery and increased cytoplasmic Munc18 puncta observed after 3 h incubation (Figure 4A–C) in treated compared to control vehicle-only cells. Munc18 was visualized via immunofluorescence, and the cells were imaged using compressed z-stacks by confocal microscopy. Munc18-positive vesicles and cells were counted via software analysis according to the inclusion and exclusion criteria described in Methods (Figure 1D,E). Experiments were conducted in triplicate and compared with controls (vehicle only). The average number of cells per field of view in the dataset was 22. Controls and treated groups were counted for the number of cells and the number of Munc18-positive vesicles based on DAPI and anti-Munc18 antibody immunostaining, respectively (Figure 4D,E). At the 3 h time point, the controls and α-syn treated groups were counted for the number of cells and number of Munc18-positive vesicles per cell based on DAPI and anti-Munc18 antibody immunostaining, respectively. Munc18-positive vesicles were significantly increased in mean number per cell after α-syn treatment (*p*, 0.001) and showed a 1.7-fold increase over controls (Figure 4F). Western blot analysis showed increased Munc18 expression in α-syn treated cells (Figure 4F, inset). As very little α-syn was observed within the cell body at 3 h incubation, being primarily at the cell periphery, we conducted an extended duration incubation with exogenous α-syn. At 24 h incubation, frequent cytoplasmic and perinuclear α-syn aggregates were observed (Figure 4G–I). The apparent slow uptake of exogenous α-syn is consistent with previous findings that uptake by primary rat astrocytes was slow in comparison with oligodendrocytes [35]. Occasional colocalization was observed between Munc18 puncta and α-syn (Figure 4C,I, arrows). Thus, exogenous WT α-syn treatment led to an activated phenotype as determined by extended morphology and increased GFAP expression (Figure 2A–F), as well as increased IL–6 secretion (Figure 4F) and increased Munc18-positive vesicles (Figure 4A–E) in 1321N1 cells, indicative of a secretory phenotype.

### 3.4. α-Syn Treatment Results in a Neuroinhibitory Astroglial Phenotype

**Figure 5:**
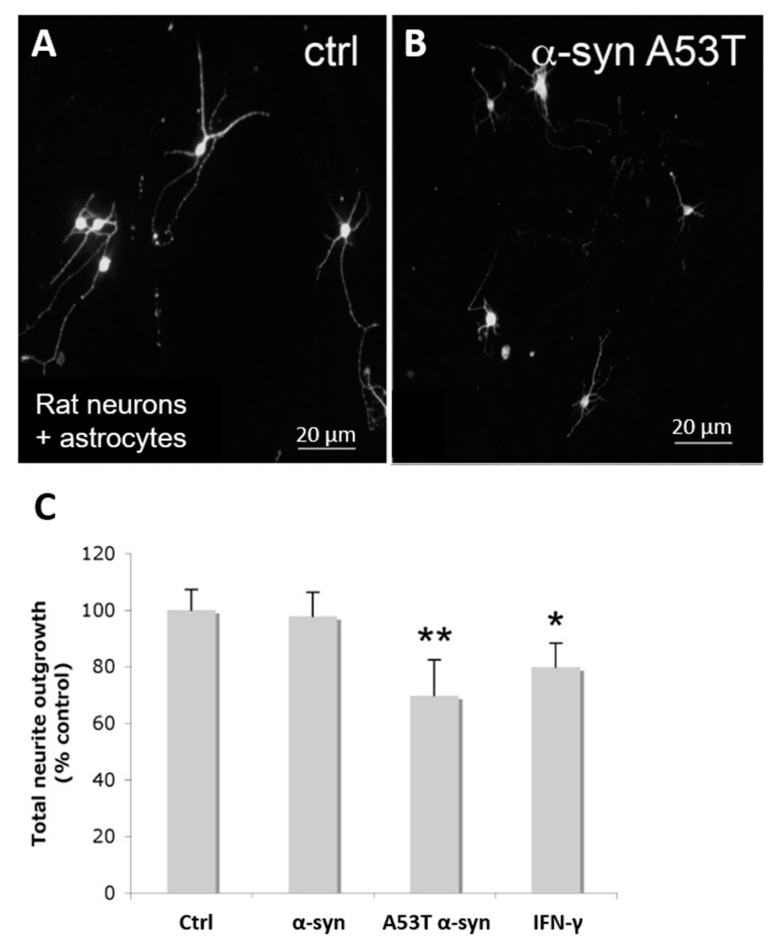
Neuron and astrocyte co-culture show astrocyte activation and neuronal inhibition. (**A**,**B**) Co-culture of rat primary neurons and astrocytes without (**A**) and with α-syn A53T pretreatment of astrocytes (**B**). (**C**) Quantification of neurite outgrowth of neurons plated onto control, α-syn WT, α-syn A53T and IFNγ pretreated astrocytes. **, *p*, 0.01; *, *p*, 0.05.

We next sought to assess the functional significance of the changes to astrocytes induced by extracellular α-syn on neuronal growth. In previous studies, the human SH-SY5Y neuroblastoma cell line has been utilized to model neurons [40,41]. However, we reverted to a rat primary co-culture model, as it was not possible to seed SH-SY5Y cells onto 1321N1 cells. Confluent astrocytes were treated with exogenous A53T α-syn for 24 h, followed by seeding of cortical neurons in the culture. After a further 24 h, the cells were fixed, and immunostaining was performed using the neuronal cytoskeletal marker βIII-tubulin. Vehicle pretreated astrocytes provided a permissive substrate to neurite outgrowth and elongation (Figure 5A). However, astrocytes pretreated with exogenous A53T α-syn provided a less-permissive substrate with neurons extending shorter neurites being much finer in caliber (Figure 5B). Quantification of neurite outgrowth demonstrated that astrocytes pretreated with exogenous A53T α-syn significantly (*p*, 0.01) impaired neurite outgrowth compared to vehicle or wild-type α-syn treated astrocytes (Figure 5C). There was no statistical difference in the number of neurons present in any of the different treatment conditions. 

## 4. Discussion

Extracellular α-syn is a potent mediator of astrocyte activation and may lead to upregulation of the secretory pathway and secretion of neuroinhibitory molecules, which can play an important role in the pathogenesis of α-synucleinopathies, including MSA and DLB [13]. We sought to investigate the hypothesis that Munc18-vesicles may be increased in activated astrocytes consistent with a secretory phenotype in MSA and DLB brain tissue compared to normal controls.

We further aimed to determine if α-syn treatment of a human astroglial cell line would result in an increased number of Munc18-positive vesicles concomitant with activation. We conducted immunostaining experiments and quantitative and semi-quantitative analysis of Munc18-positive astrocytic vesicles in MSA, DLB and normal brain tissue sections and an astrocytoma cell culture model. Intending to simulate part of the neuroinflammatory molecular milieu that occurs in neurodegenerative diseases, we used purified WT α-syn to induce morphological reactivity and functional changes in the 1321N1 human astrocytoma cell line. We hypothesized a possible role for Munc18-positive vesicles in neuroinflammation as Munc18 regulates vesicle docking in exocytosis. Previous data has revealed prominent Munc18 immunostaining in a subset of activated astrocytes in brain tissue sections of MSA cases [35]. Here, we used immunofluorescence to determine Munc18-positive vesicles in human 1321N1 cells treated with exogenous α-syn monomer, which were significantly increased in number compared to untreated. Although we have focused in the current study on treatments with exogenous α-syn monomer, granular accumulations of α-syn were observed initially at the cell periphery that may indicate aggregation of the protein in the cell culture medium or at the cell surface. It will be important in future studies to examine the possible differential effects on astrocytes of various preaggregated, prefibrillar, fibrilar and oligomeric α-syn species that have been implicated in α-synucleinopathy diseases.

Treatment with exogenous WT α-syn resulted in increased GFAP expression, activated morphology, upregulation of the secretory pathway and increased IL-6 secretion of human astroglial cells. Previously, although both WT and disease-linked mutant α-syn forms resulted in activated morphology of primary rat astrocytes, only the mutant form gave rise to statistically significant increases in GFAP immunofluorescence [35]. In the current study, GFAP ELISA assays showed increased GFAP expression of primary rat astrocytes with exogenous human α-syn, although WT α-syn again failed to reach significance. These data may indicate that rat astrocytes respond less potently than human cells to exogenous human α-syn. Previously, α-syn has been found to cause activation by interaction with the TLR-4 pathway [16,42], and subsequent phosphorylation of ERK1/2 which could be blocked by MAPK inhibition [35]. Here, extracellular α-syn-mediated promotion of a secretory astrocyte phenotype preceded α-syn uptake into the cell body, consistent with signalling via cell surface receptor.

The current study has demonstrated that Munc18-positive vesicles are increased quantitatively in response to α-syn. Moreover, immunofluorescence indicated that there was a greatly increased number of Munc18-positive vesicles in the MSA and DLB tissue compared to normal tissue, associated with activated astrocytes. Further studies will be needed to determine whether these activated astrocytes have an A1 (proinflammatory/toxic) phenotype [43] and if Munc18-positive vesicles could contribute to neurotoxicity. From the analysis of brain tissue of diseased and normal control cases of DLB and MSA, it was determined that vesicles immunopositive for Munc18, a generalized vesicle marker, are more abundant close to areas of α-syn aggregates and astrogliosis. Upon activation, exocytosis of a variety of factors by astrocytes including IL-6 is known to promote a proinflammatory environment. The precise role of the Munc18-positive vesicles observed in activated astrocytes in MSA and DLB will require further study with appropriate markers to specific endomembrane compartments. Thus, as Munc18 associates with vesicles docked for fusion with the plasma membrane and exocytosis, it may represent an objectively measurable quantitative molecular marker of neuroinflammation in α-synucleinopathies.

Coupling neuroinflammation and vesicle exocytosis apparatus, expression of Munc18 has been shown to be inducible by cytokines [44], one of which, IL-6, is primarily produced by astrocytes in the CNS [45,46]. IL-6 is a potent proinflammatory molecule present at low levels during homeostatic conditions, but induced by pathogens, trauma and neurodegeneration [47,48,49]. Indeed, IL-6 has been found to be elevated in the serum and CSF of MSA patients [50]. Moreover, astroglial treatment with exogenous WT α-syn resulted in increased IL-6 secretion.

Furthermore, exogenous α-syn treatment was found to result in the increased astrocytic expression of CSPG and a neuroinhibitory phenotype. Although only exogenous A53T α-syn gave statistically significant neuroinhibition with rat primary astrocytes, human astrocytes did show potent activation and upregulation of the secretory pathway with WT α-syn, so we cannot exclude the possibility that human astrocytes may respond more potently than rat astrocytes to the human α-syn protein in terms of neuroinhibition. Future studies could overcome these limitations by utilizing differentiated human induced pluripotent stem cell (iPSC) derived cell types [51].

Recent studies have implicated microglia as an intermediary in between extracellular α-syn and astrocyte activation [52]. Our current and previous studies show exogenous α-syn can mediate astrocyte activation independent of microglia. In the case of primary astrocyte culture, the astrocytes are grown to confluence first and at this point astrocyte division has ceased due to contact inhibition. Microglia, however, start to proliferate rapidly at this point. Ara-C is added for two days after confluence to reduce any proliferative (non-astrocytic) cells that are present in the culture.

Disturbance of the vesicle trafficking system has been implicated in the pathogenesis of PD [recently reviewed in [53]). Taken together, the current results indicate that the secretory machinery is significantly upregulated in the astrocyte response to extracellular α-syn and may participate in the release of neuroinhibitory factors in α-synucleinopathies such as PD, MSA and DLB. Further studies will be required to delineate the signaling mechanisms that link astrocyte activation, upregulation of the secretory pathway and neuroinhibition, which may include the use of microfluidic chambers to separate cell types.

## Abbreviations

α-syn	Alpha-synuclein
CSPG	Chondroitin-sulphate proteoglycan
JNK	c-Jun N-terminal kinases
DLB	Dementia with Lewy bodies
GCI	Glial cytoplasmic inclusions
GFAP	Glial fibrillary acidic protein
iNOS	inducible NO synthase
Interferon gamma	IFNγ
IL-6	Interleukin-6
MSA	Multiple system atrophy
NF-κB	Nuclear factor of kappa light polypeptide gene enhancer in B-cells
P38 MAPK	p38 mitogen-activated protein kinase
PD	Parkinson’s disease
SNAP	Soluble NSF Attachment Protein
SNARE	SNAP receptor
TLR	Toll-like receptor
